# Using Implicit Measures of Discrimination: White, Black, and Hispanic Participants Respond Differently to Group-Specific Racial/Ethnic Categories vs. the General Category “People of Color” in the USA

**DOI:** 10.1007/s40615-022-01353-z

**Published:** 2022-07-05

**Authors:** Maddalena Marini, Pamela D. Waterman, Emry R. Breedlove, Jarvis T. Chen, Christian Testa, Dana J. Pardee, Merrily LeBlanc, Sari L. Reisner, Apriani Oendari, Nancy Krieger

**Affiliations:** 1 Istituto Italiano Di Tecnologia, Via Fossato di Mortara, 19, 44121 Ferrara, FE, Italy; 2 Dipartimento Di Psicologia, Universitá Degli Studi Della Campania “Luigi Vanvitelli”, Viale Ellittico, 31, 81100 Caserta, Italy; 3 Harvard T.H. Chan School of Public Health, Boston, MA, USA; 4 The Fenway Institute, Boston, MA, USA; 5 Center for Community Health Education Research and Service, Boston, MA, USA

**Keywords:** Discrimination, Implicit measures, Implicit Association Test (IAT), Brief Implicit Association Test (B-IAT), Race/ethnicity

## Abstract

Recent studies showed that implicit measures are valuable instruments for assessing exposure to discrimination and predicting negative physical conditions. Between March 10, 2020, and April 1, 2020, we conducted three experiments (577 participants) in the USA to evaluate the use of group-specific vs. general race/ethnicity categories in implicit measures of discrimination. We measured implicit discrimination and attitudes towards the general race/ethnicity category “people of color” (POC) and two specific race/ethnicity categories (i.e., “Black people” and “Hispanic people”). Implicit discrimination and attitudes were assessed using the Brief Implicit Association Test (B-IAT). Among participants (mean age = 37, standard deviation = 10.5), 50% identified as White non-Hispanic (NH), 33.3% as Black NH, and 16.7% as Hispanic; 71.7% were female and 72.2% had a bachelor’s degree or higher. We found an implicit discrimination towards target groups and an in-group preference among all participant groups only when specific race/ethnicity categories were used in the B-IAT. When the general category POC was used, we observed a discrimination towards POC only for Black NH participants, while White NH participants showed no discrimination. Similarly, Black NH participants showed no in-group preference for POC, but did show an in-group preference for Black people. These results suggest that using the category POC in implicit measures may be inappropriate when evaluating discrimination and attitudes towards Black and Hispanic individuals as it may not capture specific experiences of discrimination and identity in these groups.

In the USA, studies demonstrate the harmful effects of racial discrimination on physical and mental health [1, 2]. To prevent and mitigate its potential harms, it is thus crucial for health research to develop measures to quantify exposure to discrimination based on race/ethnicity.

Studies commonly rely on explicit self-reported measures of discrimination (explicit discrimination) [1, 2], leaving gaps in our understanding of implicit discrimination experiences, which reflect conscious and controllable evaluations that are subject to intentional and social desirability processes [3]. Explicit measures, however, are not designed to assess what can be measured by implicit measures, which are believed to infer automatic and spontaneous mental representations that exist in memory [4], and thus to capture constructs that are outside of intentional and direct control.

Research has demonstrated that implicit and explicit measures can display a large degree of dissociation or even opposite effects [5–7]. Moreover, studies have shown that implicit measures not only can predict variations in diverse behaviors, but they can do so often with more accuracy than explicit measures [8–11]. Of note, a series of health studies using the Implicit Association Test (IAT) [12]—a widely used and validated implicit measure—showed that a greater implicit recognition of racial discrimination towards Black people is positively associated with smoking [13], elevated blood pressure, and risk of hypertension [14, 15].

In a new study to evaluate use of implicit measures for assessing multiple types of discrimination in health and other population research [16], we have shown that a new brief validated version of the IAT (i.e., the Brief IAT, B-IAT) [17, 18] is a valuable instrument for quickly assessing discrimination based on race/ethnicity, sex, gender identity, sexual orientation, age, and weight. The B-IAT developed detected an implicit recognition of discrimination towards all target groups, among both individuals belonging to the specified target and perpetrator groups. However, one notable exception emerged: in the experiment assessing discrimination based on race/ethnicity, White individuals showed no implicit discrimination towards people of color (POC) [16].

One potential explanation of this result may be related to the term POC. In the USA, POC is primarily used to describe any person who is not considered White, e.g., African Americans, Asian Americans, Native Americans, Pacific Islander Americans, multiracial Americans, and Latino Americans. This term was introduced in the USA in the late twentieth century to counter the condescension implied by the terms “non-White” and “minority” [19]. However, some have argued that the term POC lessens the focus on the specific types of racism directed towards diverse racialized groups in the USA [20], particularly Black Americans [21], for whom the term POC does not capture the specificity of anti-Black racism. It thus is plausible to hypothesize that whereas White individuals may show implicit discrimination towards specific race/ethnicity groups (e.g., Black Americans), they might not for the term “POC” because of its lack of specificity [22, 23]. To test this hypothesis, we present a study in which we investigated the impact of using general versus specific race/ethnicity categories in implicit and explicit measures of discrimination. We also employed implicit and explicit measures of racial attitudes (i.e., preferences) to test a potential relationship between discrimination and attitudes based on race/ethnicity.

## Materials and Methods

### Participants

The present study included three experiments. The People of Color-White experiment evaluated being a target vs. perpetrator of discrimination towards POC; the Black-White experiment evaluated such discrimination towards Black people; and the Hispanic-White experiment evaluated such discrimination towards Hispanic people. Between March 10, 2020, and April 1, 2020, a total of 577 participants took part in the present study and completed at least one measure included in each experiment: 199 participants in the People of Color-White experiment, 198 participants in the White-Black experiment, and 180 participants in the Hispanic-White experiment.

Participants in all three experiments were United States (U.S.) citizens and residents, aged between 25 and 64 years. This age range was selected to focus on working-age adults, given the documented importance of work as a primary site for exposure to discrimination [1, 24–26] and with this age group also having had the possibility of experiencing discrimination in multiple domains (e.g., at school, getting housing, from the legal system or police, getting medical care, getting a loan or mortgage, shopping, on the street or in a public setting) [1, 24, 25, 27–31].

Participants in each experiment were recruited based on their self-identified race/ethnicity. In the People of Color-White experiment and in the Black-White experiment, only White non-Hispanic (NH) and Black NH participants were recruited, while in the Hispanic-White experiment, we enrolled only White NH and Hispanic participants. In the People of Color-White experiment, we enrolled only Black NH participants to test whether the term POC specifically obscures the deep animus of anti-Black racism [21].

All data were collected among volunteers at the Project Implicit website (https://implicit.harvard.edu), where they first completed a demographic registration form in which they were asked their self-identified race/ethnicity, age in years, sex assigned at birth, education, citizenship, and residency. Black NH participants were then randomly assigned to the People of Color-White or Black-White experiments; Hispanic participants were assigned to the Hispanic-White experiment; and White NH were randomly assigned to People of Color-White, Black-White, or Hispanic-White experiments. Once assigned to one of our experiments, participants completed an additional race/ethnicity item in which they evaluated themselves as a person of color or a White NH person (see response options for all sociodemographic questions in Table S1).

Data collection stopped once each experiment reached 150 participants (i.e., 75 participants for each race/ethnicity group of interest) with completed measures. This sample size was defined a priori to have a greater than 90% power to detect an implicit or an explicit score significantly different from zero with an effect size of Cohen’s *d* = 0.25 and an alpha significance criterion of 0.05 (one-tailed *t* test; G ∗ Power 3) [32].

The study protocol was approved by the institutional review board at the Harvard T.H. Chan School of Public Health (IRB 18–1128).

### Brief Implicit Association Test

The Brief Implicit Association Test (B-IAT) is a new validated version of the IAT [12] that assesses mental contents (i.e., the contents of a mental state, such as a thought, a belief, or an attitude) indirectly by measuring how quickly and accurately a person can categorize and associate stimuli related to two conceptual categories and two evaluative attributes. The underlying presumption is that categories and attributes that are strongly associated at a mental representation level show shorter latencies and fewer errors when classified together than when they are not [12, 33]. Comparing latencies and errors in different experimental conditions is a conventional procedure in cognitive and social psychology for reaction time–based paradigms, such as the IAT and its versions, to infer the content of a mental state. Implicit mental content is operationalized by hypothesizing that if two concepts are mentally connected, it will be easier for participants to match these two concepts by using the same motor response compared to when they are not. This advantage in the performance will translate in faster latencies and fewer errors in the task.

The IAT has been used in hundreds of published research studies and has amassed a large literature clarifying its psychometric properties [34–37]. Studies demonstrate the IAT has excellent internal validity [36, 38–40], substantial internal consistency [41–43], stable test–retest reliability over time [33], and good construct validity [44–47].

Specifically, two B-IATs were used: a Target/Perpetrator Brief Implicit Association Test (Target/Perpetrator B-IAT) to assess implicit discrimination towards the target group and a Good/Bad Brief Implicit Association Test (Good/Bad B-IAT) to assess implicit racial/ethnic attitudes (i.e., preferences for a specific race/ethnicity group). In both B-IATs, participants were presented with words belonging to two attributes and two categories. Attributes in the Target/Perpetrator B-IAT and Good/Bad B-IAT were the same in all the experiments. The Target/Perpetrator B-IAT included words from the two attributes Target of Discrimination (e.g., victim and oppressed) and Perpetrator of Discrimination (e.g., perpetrator and abuser), while the Good/Bad B-IAT included words from the two attributes Good (e.g., love and pleasant) and Bad (e.g., hate and unpleasant). Categories in the Target/Perpetrator B-IAT and Good/Bad B-IAT differed by experiment. In the People of Color-White experiment, Target/Perpetrator B-IAT and Good/Bad B-IAT included words from the two race/ethnicity categories People of Color (e.g., Brown and non-White) and White People (e.g., White and Caucasian, with the latter problematic term chosen deliberately for reasons explained in Table 1); in the Black-White experiment, Target/Perpetrator B-IAT and Good/Bad B-IAT included words from the two race/ethnicity categories Black People (e.g., Black and African Descent) and White People (e.g., White and Caucasian); and in the Hispanic-White experiment, Target/Perpetrator B-IAT and Good/Bad B-IAT included words from the two race/ethnicity categories Hispanic People (e.g., Latinx and Hispanic) and White People (e.g., White and Caucasian). Specific stimuli used in the B-IATs are reported in Table 1. Both B-IATs followed the standard task procedure described by Sriram and Greenwald (2009). Participants were presented with words belonging to two attributes and two categories. They were instructed to focus on just one category and one attribute. Stimuli appeared one at a time in the middle of the screen and participants categorized each stimulus as either belonging to one of the focal category or attribute (press the “i” key) or not (press the “e” key). If the participant made an error, a red “X” appeared below the stimulus and the trial continued until the correct key was pressed.

Each B-IAT included four blocks of 20 trials each. In each block, the first four trials were selected from the race/ethnicity categories of interest (e.g., Black People and White People in the Black-White experiment). The remaining 16 trials for each block alternated between race/ethnicity categories of interest and attributes (i.e., Target of Discrimination and Perpetrator of Discrimination for the Target/Perpetrator B-IAT; Good and Bad for the Good/Bad B-IAT). All the blocks had the same focal attribute (i.e., Target of Discrimination for the Target/Perpetrator B-IAT and Good for the Good/Bad B-IAT) and alternated the focal category (Black People and White People in the Black-White experiment) such that the same combination between attribute and focal category (e.g., Black People+Target of Discrimination or Black People + Good) appeared in blocks 1 and 3 and the other combination (e.g., White People + Target of Discrimination or White People + Good) in blocks 2 and 4. The order of the combinations between attribute and focal category was counterbalanced across subjects.

Target/Perpetrator B-IAT and Good/Bad B-IAT were preceded by two 20-trial warm-up blocks. In one block, participants were presented with flowers (e.g., orchid and lilac) and good words as the focal categories and insects (e.g., flea and centipede) and bad words as non-focal categories. In the other block, participants were presented with insects and good words as the focal categories and flowers and bad words as non-focal categories. The order of the two practice blocks was counterbalanced across subjects.

Scores for the Target/Perpetrator B-IAT and Good/Bad B-IAT were computed according to the recommended B-IAT algorithm described by Nosek et al. (2014). We divided the difference in mean between the two B-IAT attribute-focal category conditions by the standard deviation of the latencies inclusive of the two conditions. Responses in the first four trials of each block and those slower than 10,000 ms were removed. Responses faster than 400 ms or slower than 2000 ms were recoded to 400 ms and to 2000 ms, respectively.

Participants faster than 400 ms on more than 10% of trials were excluded as indicative of careless participation. 8.5% of the B-IAT sessions were removed based on these exclusion criteria.

Scores could range from + 2 to − 2, with zero indicating no difference in association between attributes and social categories.

### Self-Reported Items

In all experiments, explicit discrimination and attitudes were measured using self-reported items (see specific questions and responses employed in Table S2).

Explicit discrimination was assessed by two questions: one evaluating whether participants perceived the group as a target of discrimination (explicit group discrimination; e.g., in the Black-White experiment, “How often do you feel that Black people are discriminated against because of their race/ethnicity?”) and one assessing whether they themselves as individuals had experienced discrimination (explicit individual discrimination; e.g., in the Black-White experiment, “How often do you feel that you, personally, have been discriminated against because of your race, ethnicity, or color?”). Responses were obtained on a 4-point scale and coded as scores from 0 (never) to 3 (often).

Explicit attitudes were measured using a single item. Participants were asked to select which statement best described them from seven options. For example, in the Black-White experiment, options were as follows: (1) I strongly, (2) I moderately, (3) I slightly “prefer Black People to White People,” (4) “I like Black people and White people equally,” (5) I slightly, (6) I moderately, and (7) I strongly “prefer White people to Black people.” Responses were coded as scores from − 3 to + 3 with more positive scores indicating an explicit preference for White people.

### Procedure

Given that the primary interest of this research was in implicit cognition, the order of the measures for each type of discrimination was fixed. For each type of discrimination, participants first completed the two BIATs and then the two explicit measures. However, to avoid order effects, the order within implicit and explicit measures was randomized across participants. Each experiment required about 10 min to complete.

### Data Analysis

Descriptive statistics characterized participants’ demographics. One-sample *t* tests determined whether the mean explicit and implicit scores were statistically different from zero. Pearson’s correlation coefficient assessed the relationship between implicit and explicit measures and between each measure.

## Results

### Sample Characteristics

The average age of all participants was 37 (*SD* = 10.5); 50% were White non-Hispanic people, 33.3% were Black NH, and 16.7% were Hispanic; 71.7% were females and 28.2% were males; 0.7% had some or less than high school education, 3.6% had a high school degree, 23.5% had some college education, 35.7% had a bachelor of arts or a bachelor of science degree, and 36.5% had an advanced degree (e.g., graduate school, master’s degree, J.D., M.D., Ph.D., or M.B.A.). Sample demographics by experiment and participant group are presented in Table 2.

### Implicit Discrimination

Table 3 presents the implicit and explicit scores and their correlations by experiment and participant group. Overall, participant groups showed an implicit discrimination towards the target group in each experiment (i.e., POC, Black, or Hispanic). In the Black-White experiment, both White (*M* = 0.23, 95% CI [0.16, 0.30]) and Black (*M* = 0.41, 95% CI [0.32, 0.50]) participants showed *Black People* + *Target of Discrimination/White People* + *Perpetrator of Discrimination* associations. These associations were stronger for Black than White participants (*F*(1, 132) = 9.773, *p* < 0.01, *ηp*^2^ = 0.69). Similarly, in the Hispanic-White experiment, both White (*M* = 0.18, 95% CI [0.08, 0.27]) and Hispanic (*M* = 0.31, 95% CI [0.31, 0.41]) participants showed *Hispanic People* + *Target of Discrimination/White People* + *Perpetrator of Discrimination* associations. The only exception was observed in the People of Color-White experiment. In this experiment, evidence of detecting an implicit discrimination towards People of Color (i.e., *People of Color* + *Target of Discrimination/White People* + *Perpetrator of Discrimination* associations) occurred only among Black participants (*M* = 0.41, 95% CI [0.31, 0.51]), whereas White participants were neutral (*M* = 0.06, 95% CI [− 0.03, 0.16]).

### Explicit Group Discrimination

Similar to the implicit data, explicit measures showed a recognition of discrimination towards the target group for all participant groups in each experiment (Table 3). Black and White participants both reported feeling that target groups are discriminated against because of their race/ethnicity (Black-White experiment: Black participants, *M* = 2.77, 95% CI [2.65, 2.90]; White participants, *M* = 2.50, 95% CI [2.36, 2.64]; People of Color-White experiment: Black participants, *M* = 2.64, 95% CI [2.49, 2.78]; White participants, *M* = 2.74, 95% CI [2.63, 2.85]); the same held in the Hispanic-White experiment (Hispanic participants, *M* = 2.68, 95% CI [2.55, 2.81], White participants, *M* = 2.59, 95% CI [2.45, 2.74]). However, in the Black-White experiment, the Black participants were more likely than the White participants to report that Black people are exposed to discrimination (*F*(1, 144) = 8.151, *p* < 0.01, *ηp*^2^ = 0.54).

### Explicit Individual Discrimination

In all experiments, participant groups self-reported exposure to discrimination towards themselves, i.e., reported an explicit individual discrimination. Black and White participants both reported feeling personally discriminated against because of their race/ethnicity (Black-White experiment: Black participants: *M* = 2.15, 95% CI [1.99, 2.31]; White participants: *M* = 0.94, 95% CI [0.79, 1.10]; People of Color-White experiment: Black participants: *M* = 2.08, 95% CI [1.89, 2.27]; White participants: *M* = 0.77, 95% CI [0.60, 0.93]); the same held for the Hispanic-White experiment (Hispanic participants: *M* = 1.70, 95% CI [1.51, 1.89]; White participants: *M* = 0.86, 95% CI [0.68, 1.03]). However, compared to White participants, members of the target groups (i.e., Black NH and Hispanic participants) reported more exposure to discrimination towards themselves (Black-White experiment: *F*(1, 144) = 116.979, *p* < 0.001, *ηp*^2^ = 0.45; People of Color-White experiment: *F*(1, 146) = 107.548, *p* < 0.001, *ηp*^2^ = 0.42; Hispanic-White experiment: *F*(1, 134) = 42.181, *p* < 0.001, *ηp*^2^ = 0.24).

### Implicit-Explicit Discrimination Correlations

Few statistically significant correlations were observed between implicit and explicit discrimination scores (Table 3). In the People of Color-White experiment, Black participants who registered stronger implicit discrimination towards People of Color also reported experiencing greater explicit individual discrimination (*r* = 0.29, *p* < 0.05). In the Hispanic-White experiment, White participants who showed stronger implicit discrimination towards Hispanic people reported weaker explicit individual discrimination (*r* = − 0.26, *p* < 0.05).

### Implicit Attitudes

Overall, participant groups showed an implicit in-group attitude (Table 3). White participants showed a preference for White people (Black-White experiment: *M* = 0.21, 95% CI [0.11, 0.31]; Hispanic-White experiment: *M* = 0.10, 95% CI [0.01, 0.19]), Black participants showed a preference for Black people (Black-White experiment: *M* = − 0.23, 95% CI [− 0.33, − 0.13]), and Hispanic participants showed a preference for Hispanic people (Hispanic-White experiment: *M* = − 0.22, *SD* = 0.37, 95% CI [− 0.31, − 0.13]). However, in the People of Color-White experiment, an in-group attitude was observed only for White participants (*M* = 0.33, 95% CI [0.23, 0.43]), while Black participants showed no implicit preference for People of Color or White people (*M* = − 0.09, 95% CI [− 0.21, 0.02]).

### Explicit Attitudes

Results for explicit attitudes varied by experiment. Black (Black-White experiment: *M* = − 1.37, 95% CI [− 1.66, − 1.08]) and Hispanic (Hispanic-White experiment: *M* = − 0.70, 95% CI [− 0.99, − 0.41]) participants reported an in-group attitude, while White participants reported no significant attitudes (Black-White experiment: *M* = 0.03, 95% CI [− 0.13, 0.19]; Hispanic-White experiment: *M* = 0.05, 95% CI [− 0.16, 0.27]). Conversely, in the People of Color-White experiment, both Black (*M* = − 1.34, 95% CI [− 1.64, − 1.05]) and White (*M* = 0.24, 95% CI [0.09, 0.39]) participants reported an explicit in-group attitude.

### Implicit-Explicit Attitude Correlations

No statistically significant correlations between implicit and explicit attitudes emerged in experiments.

### Implicit and Explicit Discrimination and Attitude Measures Correlations

Significant negative correlations between implicit discrimination and attitudes were observed for Black participants in the People of Color-White experiment (*r* = − 0.45, *p* < 0.001) and for Hispanic participants in the White-Hispanic experiment (*r* = − 0.29, *p* < 0.05) (Table S3), indicating that for these participants, stronger implicit discrimination towards the target group was associated with a weaker implicit preference for White people. A significant negative correlation between explicit group discrimination and explicit attitudes emerged for White participants in the Black-White experiment (*r* = − 0.29, *p* < 0.05), indicating that White participants who reported stronger explicit discrimination towards Black people showed weaker explicit preferences for White people. Additionally, in the People of Color-White experiment, Black participants who showed weaker implicit preferences for White people reported more exposure to discrimination towards themselves (*r* = − 0.25, *p* < 0.05), whereas White participants who showed weaker implicit discrimination towards People of Color reported stronger explicit preferences for White people (*r* = − 0.26, *p* < 0.05).

## Discussion

The present study investigated the effect of using specific versus general race/ethnicity categories in implicit and explicit measures of discrimination in the U.S. The central finding was that an implicit discrimination towards target groups was detected among all participants only when specific race/ethnicity categories were used in the B-IAT (i.e., in the Black-White and Hispanic-White experiments, but not in the White-People of Color experiment). The larger implication is that using specific race/ethnicity categories in implicit measures of discrimination may be more appropriate than using the general category POC.

Supporting our interpretation that POC fails to elicit the same response as the specific terms “Black” and “Hispanics” among White NH persons is the dramatic rise, since 2020, of the term “BIPOC” in the U.S. [51–55]. This acronym stands for “Black, Indigenous, and People of Color” and it was coined in part because the term “POC” failed to capture the different types of racism experienced by diverse racialized groups in the U.S., including the excessive use of police violence targeted especially against Black and Indigenous persons [51–55]. Although POC was introduced in the U.S. in the late twentieth century (around the late 1970s) [56, 57] to counter the terms “minority” and “non-White,” which were believed to reflect marginalization and racism, critics of the term have objected to its lack of specificity and find it racially offensive [22, 23] as it lessens the focus on distinct issues confronted by different racial and ethnic groups [20], particularly African Americans [21]. Preserving “whiteness” as an intact category while lumping every other racial group into an indiscriminate category (“of color”) can replicate the very marginalization the term was intended to counter. While the term “BIPOC” is intended to address this concern, the continued lumping of all racialized groups other than Black, Indigenous, and White into the “POC” part of “BIPOC” remains contested, acceptable to some, and rejected by others [51–55]. Future research comparing how members of diverse racialized groups respond, both implicitly and explicitly, to the terms “BIPOC” and “POC” would be informative.

In addition, we also found that implicit attitudes showed different results across participant groups when the term “POC” or “Black people” was used. Black people showed no in-group preference for POC, but did show an in-group preference for Black people. This result suggests that Black people may not identify themselves as members of the group POC at the implicit level even though they reported considering themselves as people of color and showed an explicit in-group attitude on the explicit measures. This result seems to be in contrast with POC racial identity theory [58, 59], which states that although a person’s experience of racism may vary, POC develop a common racial identity as individuals who experience similar psychological struggles, coping strategies related to being classified, treated, and perceived as a person who is not white. This race identity develops regardless of nationality, language, or culture of origin. As suggested, however, by the rising use of the term BIPOC, the premises of this version of racial identity theory may no longer hold. Future studies, which measure both implicit and explicit race/ethnicity identity, and which take into account new shifts in terminology, would be valuable to test the extent to which the original POC racial identity theory does or does not hold among diverse racialized groups in the U.S. and potentially in other countries as well.

The findings of this study should be considered in light of its limitations. Although the sample was statistically large enough to make inferences about a population, it is not a random selection of the U.S. population. For example, 72.2% of our study’s participants had a bachelor’s degree or higher, which is fully two times higher than reported for U.S. adults age 25 and older in 2019 [60, 61]. In addition, it included a much larger number of female (71.7%) than male (28.2%) participants. However, we cannot identify a specific and valid reason why these factors would explain the different results observed in implicit measures when the term POC or specific race/ethnicity categories were used. Nonetheless, replication with other samples will be useful to increase confidence in the observed results. Such research also should be conducted in populations at diverse educational levels and recruiting a larger number of male participants.

In sum, our findings show that language can influence the recognition of discrimination towards target groups at the implicit level. In particular, we showed that the term POC may be too broad to capture the specific experience of racial discrimination for Black Americans and masks the discrimination that White Americans may direct specifically and distinctly at Black Americans compared to other racialized groups. These findings may have important implications for health policy and practice in the clinical setting. For example, they raise the question of whether it is appropriate or not to use the general term POC when referring to Black patients or to patients in other racialized groups included under the umbrella term POC. Possible harms may include blunting recognition, by White health care professionals and by the patients themselves, of the specific types of discrimination directed against specific racialized groups, thereby potentially exacerbating inequities in both health status and health care.

## Supplementary Material

1821956_Sup_Tab_1

1821956_Sup_Tab_2

1821956_Sup_Tab_3

## Figures and Tables

**Table 1:** Stimuli used in the B-IATs by experiment

B-IAT	B-IAT attributes	Stimuli
Target/Perpetrator	Target of Discrimination	Target, victim, oppressed
	Perpetrator of Discrimination	Perpetrator, abuser, racist
Good/Bad	Good	Love, pleasant, great, wonderful
	Bad	Hate, unpleasant, awful, terrible
Experiment	B-IAT categories	Stimuli
People of Color-White	White People	White, Euro-American, Caucasian
	People of Color	Black, Brown, non-White
Black-White	White People	White, Euro-American, Caucasian
	Black People	Black, African Descent, African American
Hispanic-White	White People	White, Euro-American, Caucasian
	Hispanic People	Latinx, Latino/a, Hispanic

Attributes in the Target/Perpetrator B-IAT and Good/Bad B-IAT were the same in all the experiments, while categories differed on the basis of the experiment. For example in the Black-White experiment, Target/Perpetrator B-IAT and Good/Bad B-IAT included words from the two categories White People (e.g., White and Caucasian) and Black People (e.g., Black and African Descent), while in the Hispanic-White experiment, Target/Perpetrator B-IAT and Good/Bad B-IAT included words from the two categories White People (e.g., White and Caucasian) and Hispanic People (e.g., Latinx and Hispanic). We chose these terms because of their common use, but acknowledge that the term “Caucasian,” while widely used, is a word that has deep roots in scientific racism and is not a legitimate scientific term or an accurate description (since it falsely conveys the idea humanity originated in the Caucasus Mountains in Europe); for more on the problematic history of the term “Caucasian,” see [48–50]

**Table 2:** Sample demographics by experiment and participant group

Experiment	Black-White		People of Color-White		Hispanic-White		Total

Participant group: demographics	White NH *N* = 89	Black NH *N* = 109	White NH *N* = 94	Black NH *N* = 105	White NH *N* = 103	Hispanic *N* = 77	*N* = 577
Sex							
% female	65.9	73.4	68.8	72.1	77.7	72.7	71.7
% male	34.1	26.6	31.2	27.9	22.3	27.3	28.2
Race/ethnicity							
% White NH	100	0	100	0	100	0	50
% Black NH	0	100	0	100	0	0	33.3
% Hispanic	0	0	0	0	0	100	16.7
Education (highest level completed)							
% some or less than high school	0.9	0	0	1.0	1.0	1.3	0.7
% high school	4.5	4.3	6.4	1.0	2.9	2.6	3.6
% some college	10.9	28.8	26.6	31.4	22.3	20.8	23.5
% BA/BS	39.1	30.9	23.4	35.2	35.0	50.6	35.7
% advanced degree	44.5	36.0	43.6	31.4	38.8	24.7	36.5
Age							
Mean	40.1	37.9	35.4	37.6	38.6	32.1	37
SD	10.7	10.5	9.9	10.5	12.1	9.2	10.5

Sample size (*N*) and demographics refer to data of participants who completed at least one of the four measures used in each experiment (i.e., Target/Perpetrator B-IAT, Good/Bad B-IAT, self-reported items assessing explicit discrimination, and self-reported item assessing explicit attitudes). Sex refers to sex assigned at birth. All participants were U.S. citizens and residents, aged between 25 and 64 years. Percentages refer to percent distribution with no missing data

**Table 3:** Implicit and explicit scores for discrimination and attitudes by experiment and participant group and their correlation

Experiment	Participant group	Implicit measures						Explicit measures									Implicit and explicit correlations		
		Target/Perpetrator B-IAT (implicit discrimination)			Good/Bad B-IAT (implicit attitude)			Explicit group discrimination			Explicit individual discrimination			Explicit attitude			Target/Perpetrator B-IAT and explicit group discrimination	Target/Perpetrator B-IAT and explicit individual discrimination	Good-Bad B-IAT and explicit attitude
							
		*N*	Mean (SD)	Group comparison	*N*	Mean (SD)	Group comparison	*N*	Mean (SD)	Group comparison	*N*	Mean (SD)	Group comparison	*N*	Mean (SD)	Group comparison	*r*	*r*	*r*
People of Color-White	White NH	69	0.06 (0.40)	**	71	0.33** (0.42)	**	73	2.74** (0.47)	n.s	73	0.77** (0.72)	**	72	0.24** (0.64)	**	− 0.09	− 0.18	0.04
	Black NH	64	0.41** (0.40)		70	− 0.09 (0.48)		74	2.64** (0.63)		75	2.08**(0.82)		70	− 1.34** (1.23)		− 0.01	0.29*	0.06
Black-White	White NH	72	0.23** (0.32)	**	73	0.21** (0.43)	**	72	2.50** (0.61)	**	72	0.94** (0.67)	**	69	0.03 (0.66)	**	0.06	− 0.11	0.24
	Black NH	62	0.41** (0.35)		71	− 0.23** (0.42)		74	2.77** (0.54)		74	2.15** (0.68)		73	− 1.37** (1.25)		0.09	− 0.08	0.09
Hispanic-White	White NH	68	0.18** (0.40)	n.s	75	0.10* (0.40)	**	76	2.59** (0.64)	n.s	76	0.86** (0.76)	**	73	0.05 (0.94)	**	− 0.02	− 0.26*	0.05
	Hispanic	57	0.31** (0.38)		61	− 0.22** (0.37)		63	2.68** (0.50)		60	1.70** (0.74)		60	− 0.70** (1.11)		− 0.17	0.24	0.01

B-IAT scores could range from + 2 to − 2, whereas explicit scores could range from 0 to + 3. Positive scores in the Target/Perpetrator B-IAT and explicit group discrimination indicated the presence of a discrimination towards the target group. Positive scores in explicit individual discrimination indicated the presence of discrimination towards self as a member of a specific social group. Positive scores in the Good/Bad B-IAT and explicit attitude indicated a preference for the dominant group. Zero indicated no relative discrimination or preference. Note: group comparison refers to comparison of scores (mean values) across the participant groups for each experiment

*M* mean, *SD* standard deviation, *n.s.* not significant

* *p* < 0.05 (2-sided)

** *p* < 0.01 (2-sided)

## Data Availability

The data of this study are available from the corresponding author upon reasonable request.
