# Supplementary material for: Using Implicit Measures of Discrimination: White, Black, and Hispanic Participants Respond Differently to Group-Specific Racial/Ethnic Categories vs. the General Category “People of Color” in the USA
Source: J Racial Ethn Health Disparities. 2022 Jul 5;10(4):1682–92. doi: 10.1007/s40615-022-01353-z (PMC9813272; doi:10.1007/s40615-022-01353-z)
Supplement: Supplementary file 1 — Supplementary file1 (DOCX 16 KB) [file 40615_2022_1353_MOESM1_ESM.docx]

| **Demographics** | **Item** |
| --- | --- |
| 1. Race/Ethnicity | What is your race/ethnicity?   - Black non-Hispanic - White non-Hispanic - Hispanic - Other or Unknown |
| 1. Sex | What is your sex at birth?   - Male - Female |
| 1. Age | How old are you?   - [response options from 1 to 99] |
| 1. Education | Please indicate the highest level of education that you have completed.   - some or less than high school education - high school degree - college education - Bachelor of Arts or Bachelor of Science degree - Advanced degree (e.g. graduate school, master’s degree, J.D., M.D., Ph.D., M.B.A.) |
| 1. Residency | What is your country of residence?   - U.S.A. - Other |
| 1. Citizenship | What is your country of citizenship?   - U.S.A. - Other |
| 1. Race/Ethnicity (people of color or White non-Hispanic person) | Do you consider yourself to be a person of color or a White, non-Hispanic person?   - Person of Color - White, non-Hispanic |

**Table S1. Demographic items used for assessing race/ethnicity, sex, age, education, residency, and citizenship.**

Note. Items from 1 to 6 were included in the demographical registration form that participants completed when they volunteered for studies at Project Implicit’s research site. Only Black NH, White non-Hispanic, Hispanic United States (U.S.) citizens and residents, aged between 25 to 64 years were enrolled in our studies. Black NH participants were randomly assigned to the People of Color-White or Black-White experiments; Hispanic participants were assigned to the Hispanic-White experiment; and White non-Hispanic (NH) were randomly assigned to People of Color-White, Black-White or Hispanic-White experiments. Once assigned to one of our experiments, participants completed an additional race/ethnicity item (item 7) in which they evaluated themselves as a person of color or a White non-Hispanic person. Respondents self-identified their characteristics at the time of the study.
