# Supplementary material for: Using Implicit Measures of Discrimination: White, Black, and Hispanic Participants Respond Differently to Group-Specific Racial/Ethnic Categories vs. the General Category “People of Color” in the USA
Source: J Racial Ethn Health Disparities. 2022 Jul 5;10(4):1682–92. doi: 10.1007/s40615-022-01353-z (PMC9813272; doi:10.1007/s40615-022-01353-z)
Supplement: Supplementary file 2 — Supplementary file2 (DOCX 26 KB) [file 40615_2022_1353_MOESM2_ESM.docx]

|  | **Item** | | |
| --- | --- | --- | --- |
| **Experiment** | **Explicit attitude** | **Explicit group discrimination** | **Explicit Individual discrimination** |
| People of Color- White | Which statement best describes you?   - I strongly prefer People of Color (e.g., Black and Hispanic) to White people. - I moderately prefer People of Color (e.g., Black and Hispanic) to White people. - I slightly prefer People of Color (e.g., Black and Hispanic) to White people. - I like People of Color (e.g., Black and Hispanic) and White people equally. - I slightly prefer White people to People of Color (e.g., Black and Hispanic). - I moderately prefer White people to People of Color (e.g., Black and Hispanic). - I strongly prefer White people to People of Color (e.g., Black and Hispanic). | How often do you feel that people of color, such as Black, and Hispanic are discriminated against because of their race/ethnicity?   - Never - Rarely - Sometimes - Often | How often do you feel that you, personally, have been discriminated against because of your race, ethnicity, or color?   - Never - Rarely - Sometimes - Often |
| Black-White | Which statement best describes you?   - I strongly prefer Black people to White people. - I moderately prefer Black people to White people. - I slightly prefer Black people to White people. - I like Black people and White people equally. - I slightly prefer White people to Black people. - I moderately prefer White people to Black people. - I strongly prefer White people to Black people. | How often do you feel that Black people are discriminated against because of their race/ethnicity?   - Never - Rarely - Sometimes - Often | How often do you feel that you, personally, have been discriminated against because of your race, ethnicity, or color?   - Never - Rarely - Sometimes - Often |
| Hispanic-White | Which statement best describes you?   - I strongly prefer Hispanic People to White people. - I moderately prefer Hispanic People to White people. - I slightly prefer Hispanic People to White people. - I like Hispanic People and White people equally. - I slightly prefer White people to Hispanic People. - I moderately prefer White people to Hispanic People. - I strongly prefer White people to Hispanic People. | How often do you feel that Hispanic people are discriminated against because of their race/ethnicity?   - Never - Rarely - Sometimes - Often | How often do you feel that you, personally, have been discriminated against because of your race, ethnicity, or color?   - Never - Rarely - Sometimes - Often |

**Table S2. Explicit items and response options by experiment.**
