# Supplementary material for: Using Implicit Measures of Discrimination: White, Black, and Hispanic Participants Respond Differently to Group-Specific Racial/Ethnic Categories vs. the General Category “People of Color” in the USA
Source: J Racial Ethn Health Disparities. 2022 Jul 5;10(4):1682–92. doi: 10.1007/s40615-022-01353-z (PMC9813272; doi:10.1007/s40615-022-01353-z)
Supplement: Supplementary file 3 — Supplementary file3 (DOCX 15 KB) [file 40615_2022_1353_MOESM3_ESM.docx]

| **Measures of Attitudes** | **Experiment** | **Participant Group** | **Measures of Discrimination** | | |
| --- | --- | --- | --- | --- | --- |
|  |  |  | **Target/Perpetrator B-IAT**  **(Implicit Discrimination)** | **Explicit Group Discrimination** | **Explicit Individual Discrimination** |
|  |  |  | r | r | r |
| **Good/Bad B-IAT (Implicit Attitude)** | **People of Color-White** | **White NH** | -0.15 | -0.13 | 0.11 |
|  |  | **Black NH** | *-0.45*** | -0.05 | *-0.25** |
|  | **Black-White** | **White NH** | -0.22 | -0.15 | 0.16 |
|  |  | **Black NH** | -0.16 | -0.05 | -0.21 |
|  | **Hispanic-White** | **White NH** | -0.10 | -0.16 | 0.08 |
|  |  | **Hispanic** | *-0.29** | 0.08 | -0.14 |
| **Explicit Attitude** | **People of Color-White** | **White NH** | *-0.26** | -0.22 | 0.06 |
|  |  | **Black NH** | 0.08 | -0.13 | -0.17 |
|  | **Black-White** | **White NH** | 0.15 | *-0.29** | -0.03 |
|  |  | **Black NH** | -0.03 | 0.09 | -0.23 |
|  | **Hispanic-White** | **White NH** | -0.10 | 0.13 | 0.20 |
|  |  | **Hispanic** | -0.22 | -0.08 | -0.22 |
| Note. *p<0.05 (2-sided); ** p<0.01 (2-sided) | | | | | |

**Table S3. Correlations between measures of discrimination and attitudes by experiment and participant group.**
